# Supplementary material for: Phylogenetic synthesis of morphological and molecular data reveals insights on the classification of diogenid hermit crabs (Crustacea: Decapoda: Anomura)
Source: PeerJ. 2024 Aug 28;12:e17922. doi: 10.7717/peerj.17922 (PMC11365476; doi:10.7717/peerj.17922)
Supplement: Supplemental Information 1 [file peerj-12-17922-s001.docx]

**Table S1:**

**The data matrix of morphological characters.**

| Family | Genus | Species | Coding for morphological characters |
| --- | --- | --- | --- |
| Lithodidae | *Paralomis* | *P. dofleini* | 0102001211----0–00011011101010000-----010 |
| Paguridae | *Pagurus* | *P. ochotensis* | 01001012110011010021111001001111100010100 |
|  |  | *P. bernhardus* | 010010021100000100211010101011111---00100 |
| Diogenidae | *Calcinus* | *C. elegans* | 01001002110101000011101101110111110011101 |
|  |  | *C. morgani* | 01001000020101000011101111100011111011101 |
|  |  | *C. latens* | 01001002120100000011101111001111110011101 |
|  |  | *C. gaimardii* | 01001002100101000011101101100011111011101 |
|  |  | *C. laevimanus* | 01001002120010000011101111111111111011101 |
|  |  | *C. vachoni* | 01001012120101000011101111100011112000101 |
|  |  | *C. guamensis* | 01001002111100000011101111101111111100101 |
|  |  | *C. minutus* | 01001000000100000011101111101011111010101 |
|  | *Clibanarius* | *C. longitarsus* | 01001002120100000001101110011011110010101 |
|  |  | *C. corallinus* | 01001002121100000001001110101011100010101 |
|  |  | *C. virescens* | 01001012121100010001101011101011110010101 |
|  |  | *C. humilis* | 01001002111101000001101110201111110000101 |
|  |  | *C. englaucus* | 01001011111100000001101110101011110011101 |
|  |  | *C. merguiensis* | 01001002121100010001101110101111100000101 |
|  |  | *C. eurysternus* | 01001012121100010001101110201011111011101 |
|  |  | *C. snelliusi* | 01001002121100010001101011211111111010101 |
|  |  | *C.infraspinatus* | 01001002121001010001101110001011111010101 |
|  |  | *C. rutilus* | 01001012121100010001101010211011111000101 |
|  | *Ciliopagurus* | *C. strigatus* | 01001010011101010000120010201111100011102 |
|  | *Dardanus* | *D. lagopodes* | 01001001101100000011111010201011100010102 |
|  |  | *D. crassimanus* | 11001001121100010011011100001011101000102 |
|  |  | *D. setifer* | 11001011121000010011111110001011111010102 |
|  |  | *D. guttatus* | 11001011121100010011011010000111101010102 |
|  |  | *D. hessii* | 01001001111000010001011011001111101010102 |
|  |  | *D. impressus* | 11001001111000010011111101001011111010102 |
|  |  | *D. gemmatus* | 11001001111000010011111011001111101000102 |
|  |  | *D. deformis* | 01001001111000010011111111001111111010102 |
|  |  | *D. arrosor* | 11001001111001010111110110001111111010102 |
|  | *Diogenes* | *D. avarus* | 00211011111001010011101111111111110110101 |
|  |  | *D. rectimanus* | 00111111111011010011111011011111110100101 |
|  |  | *D. nitidimanus* | 10211011211011010011111011011111110100101 |
|  |  | *D. edwardsii* | 00211001211011010011111011001111111100101 |
|  |  | *D. goniochirus* | 00211011111011010011101111011111110110101 |
|  | *Paguristes* | *P. doederleini* | 01011002110001100001001010001111110010011 |
|  |  | *P. miyakei* | 01011002110001110001001011001111110010011 |
|  |  | *P. albimaculatus* | 01011002110000100001101011001111101011011 |
|  |  | *P. calvus* | 01011002120100100001101010001011101011011 |
|  |  | *P. seminudus* | 01011002120100100001101011011111101011011 |
| Coenobitidae | *Coenobita* | *C. rugosus* | 11020001110000221110111011101111111011100 |
|  |  | *C. violascens* | 11020001110000221111101011111111100011100 |

**List morphological characters:**

1. Rostrum: distinct (0); absent or minute (1).

2. Rostral process: present (0); absent (1).

3. Rostral process form: absent (0); longer than ocular acicles (1); shorter than ocular acicles (2).

4. Carapace shield: unarmed or with scattered setae (0); with few scattered spines (1); densely spinose (2).

5. Posterior portion of carapace: well calcified throughout (0); partially calcified (1).

6. Anterior margin of carapace: normal (0); spinose (1).

7. Shape of shield: longer than wide (0); as long as wide (1).

8. lateral projections: reduced (0); longer than rostrum (1); equal to or shorter than rostrum (2).

9. Shape of lateral projections: reduced (0); having one spine (1); having more than one spine (2).

10. Ocualr peduncles: longer than shield (0); shorter than shield (1); as long as shield (2).

11.Ocular acicles: simple or armed with 1-2 terminal spines (0); armed with more than 3 terminal spines (1).

12. Antennular peduncles: longer than ocular peduncles (0); shorter than ocular peduncles (1).

13. Antennular peduncles: longer than antennal peduncles (0); shorter than antennal peduncles (1).

14. Antennular peduncles fourth article: unarmed (0); armed with spine (1).

15. Antennal acicle: reaching proximal margin of fifth antennal segment (0); overreaching middle of fifth antennal segment (1); reduced (2).

16. Antennal acicle shape: with doromedial spines (0); without doromedial spines (1); reduced (2).

17. Antennular upper flagellum termination: tapering (0); blunt, stick-like (1).

18. Antennal acicle: well-developed (0); fused with second article of antennal peduncle (1).

19. Chelipeds symmetry: equal or subequal (0); left distinctly larger (1); right distinctly larger (2).

20. Chelipeds: with stridulatory apparatus (0); without stridulatory apparatus (1).

21. Larger chela surface: with dense setae (0); only with tufts of setae or without setae (1).

22. Larger chela palm and dactyl: scattered with tubercles (0); with transverse corneous spines (1); without tubercles (2).

23. Chela and carpus of chelipeds: with transverse striae (0); without transverse striae (1).

24. Length of dactyl: equal to or longer than palm (0); shorter than palm (1).

25. Cutting edges of fixed finger and dactyl: with large calcareous teeth (0); without large calcareous teeth (1).

26. Terminal of fixed finger and dactyl: with clibanarius (0); without clibanarius (1).

27. Third pereopod: dactyl longer than propodus (0); dactyl shorter than propodus (1); dactyl as long as propodus (2).

28. Dactyl of ambulatory legs: with terminal corneous claw (0); without terminal corneous claw (1).

29. Left third pereopod ventral margin of dactyl and propodus: with brush-like setae (0); with sparse tufts of setae, not brush-like (1).

30. Ventral margin of dactyl of third pereopod: with spine (0); without spine (1).

31. Pereopod 4 size: normal, similar to preceding limb (0); strongly reduced (1).

32. Abdominal condition: straight (0); twisted (1).

33. Abdominal segmentation: somites distinct (0); somites ill-defined (1).

34. Posterior margin of telson: with large cleft (0); with small cleft (1).

35. Telson: with two similar posterior lobes (0); with left lobe larger than right one (1).

36. Lateral margin of telson: with cleft (0); without cleft (1).

37. Lateral margin of telson: with spines (0); without spines (1).

38. Posterior margin of telson: armed (0); unarmed (1).

39. Male paired pleopod 1: present (0); absent (1).

40. Female paired pleopods 1: absent (0); present (1).

41. Gill number: < 13 pairs (0); 13 pairs (1); 14 pairs (2).
