# Supplementary material for: Phylogenetic synthesis of morphological and molecular data reveals insights on the classification of diogenid hermit crabs (Crustacea: Decapoda: Anomura)
Source: PeerJ. 2024 Aug 28;12:e17922. doi: 10.7717/peerj.17922 (PMC11365476; doi:10.7717/peerj.17922)
Supplement: Supplemental Information 2 [file peerj-12-17922-s002.docx]

**Table S2**

**Primer sets used in PCR amplification of the four genes.**

| Primer name | Sequence (5'–3') | Reference |
| --- | --- | --- |
| 16S rRNA |  |  |
| 1471 | CCT GTT TAN CAA AAA CAT | *Crandall & Fitzpatrick, 1996* |
| 1472 | AGA TAG AAA CCA ACC TGG | *Crandall & Fitzpatrick, 1996* |
| COI |  |  |
| dgLCO1490 | GGT CAA CAA ATC ATA AAG AYA TYG G | *Meyer, 2003* |
| dgHCO2198 | TAA ACT TCA GGG TGA CCA AAR AAY CA | *Meyer, 2003* |
| NaK |  |  |
| NaK for-a | GTG TTC CTC ATT GGT ATC ATT GT | *Tsang et al., 2008* |
| NaK for-b | ATG ACA GTT GCT CAT ATG TGG TT | *Tsang et al., 2008* |
| NaK rev | ACC TTG ATA CCA GCA GAT CGG CAC TTG GC | *Tsang et al., 2008* |
| NaK rev2 | ATA GGG TGA TCT CCA GTR ACC AT | *Tsang et al., 2008* |
| NaK rev3 | GGA GGR TCA ATC ATR GAC AT | *Tsang et al., 2014* |
| PEPCK |  |  |
| PEPCK for | GTA GGT GAC GAC ATT GCY TGG ATG AA | *Tsang et al., 2008* |
| PEPCK for2 | GCA AGA CCA ACC TGG CCA TGA TGA C | *Tsang et al., 2008* |
| PEPCK rev | GAA CCA GTT GAC GTG GAA GAT C | *Tsang et al., 2008* |
| PEPCK rev3 | CGG GYC TCC ATG CTS AGC CAR TG | *Tsang et al., 2008* |

Crandall KA, Fitzpatrick JF. 1996. Crayfish molecular systematics: using a combination of procedures to estimate phylogeny. *Systematic Biology* 45(1):1–26.

Meyer CP. 2003. Molecular systematics of cowries (Gastropoda: Cypraeidae) and diversification patterns in the tropics. *Biological Journal of the Linnean Society* 79(3):401–459. DOI:10.1046/j.1095-8312.2003.00197.x

Tsang LM, Ma KY, Ahyong ST, Chan TY, Chu KH. 2008. Phylogeny of Decapoda using two nuclear protein-coding genes: Origin and evolution of the Reptantia. *Molecular Phylogenetics and Evolution* 48(1):359–368. DOI:10.1016/j.ympev.2008.04.009

Tsang LM, Schubart CD, Ahyong ST, Lai JCY, Au EYC, Chan TY, Ng PKL, Chu KH. 2014. Evolutionary History of True Crabs (Crustacea: Decapoda: Brachyura) and the Origin of Freshwater Crabs. *Molecular Biology and Evolution* 31(5):1173–1187. DOI:10.1093/molbev/msu068
