# Supplementary material for: Phylogenetic synthesis of morphological and molecular data reveals insights on the classification of diogenid hermit crabs (Crustacea: Decapoda: Anomura)
Source: PeerJ. 2024 Aug 28;12:e17922. doi: 10.7717/peerj.17922 (PMC11365476; doi:10.7717/peerj.17922)
Supplement: Supplemental Information 3 [file peerj-12-17922-s003.docx]

**Table S****3:**

**Best partitioning scheme and best-fit substitution model selected by PartitionFinder.**

| Partition | Model |
| --- | --- |
| COI 1 | GTR + I+ G |
| COI 2, NaK 2, PEPCK 2 | GTR + I+ G |
| COI 3 | GTR + G |
| NaK 1, PEPCK 1 | GTR + I+ G |
| NaK 3, PEPCK 3 | GTR + G |
| 16S rRNA | GTR + I+ G |
