# Supplementary figures and images for: Phylogenetic synthesis of morphological and molecular data reveals insights on the classification of diogenid hermit crabs (Crustacea: Decapoda: Anomura)
Source: PeerJ. 2024 Aug 28;12:e17922. doi: 10.7717/peerj.17922 (PMC11365476; doi:10.7717/peerj.17922)

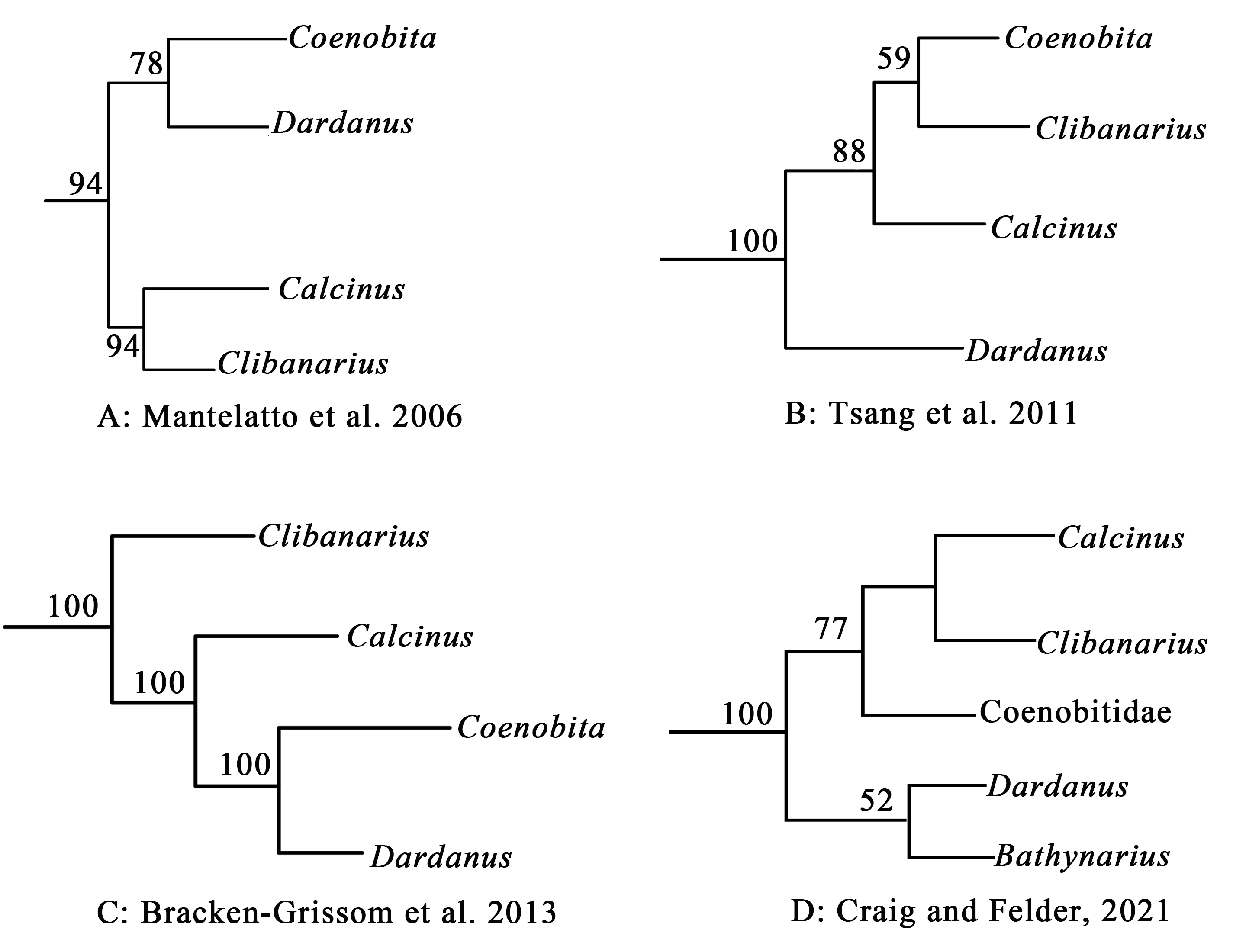

Supplement: Supplemental Information 4 [file peerj-12-17922-s004.png]

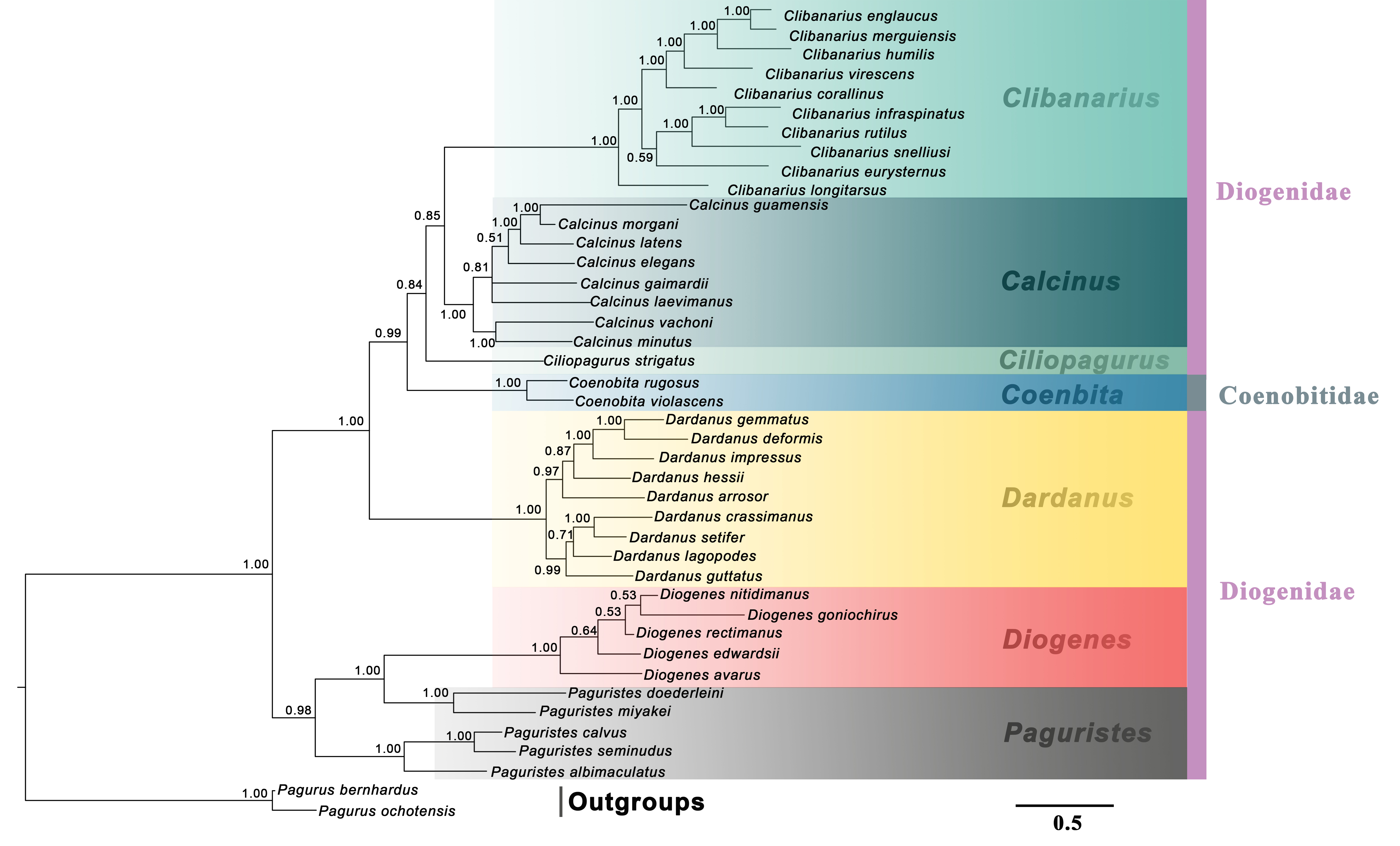

Supplement: Supplemental Information 5 — Nodal support values represent Bayesian posterior probabilities (Pp). Two Pagurus species from the family Paguridae were used to root the phylogenetic tree. [file peerj-12-17922-s005.png]
